# Newly synthesized M^pro^ inhibitors as potential oral anti-SARS-CoV-2 agents

**DOI:** 10.1038/s41392-021-00560-0

**Published:** 2021-03-31

**Authors:** Lin Li, Shile Huang

**Affiliations:** 1 Department of Biochemistry and Molecular Biology, Louisiana State University Health Sciences Center, Shreveport, LA USA; 2 Department of Hematology and Oncology, Louisiana State University Health Sciences Center, Shreveport, LA USA; 3 Feist-Weiller Cancer Center, Louisiana State University Health Sciences Center, Shreveport, LA USA

**Keywords:** Molecular medicine, Target validation

In a recent paper published in *Science*, Qiao et al.^1^ described the design, synthesis, and characterization of 32 new SARS-CoV-2 M^pro^ inhibitors. SARS-CoV-2 M^pro^ inhibitors have been reported,^2^ but there is no infection data in any animal models. For the first time, Qiao et al.^1^ demonstrated that oral or intraperitoneal treatment with two compounds (MI-09 and MI-30) exhibits effective antiviral activity in a SARS-CoV-2 infection transgenic mouse model.

COVID-19, due to SARS-CoV-2 infection, has emerged as a global pandemic, causing high morbidity and mortality. To combat COVID-19, diverse COVID-19 vaccines have been developed, showing positive results in preventing SARS-CoV-2 infection. However, so far, only remdesivir, a broad-spectrum antiviral drug, has been approved or authorized for emergency use to intravenously treat patients with COVID-19 in about 50 countries, despite having adverse effects.^3^ Thus, it is imperative to develop specific anti-SARS-CoV-2 drugs with better safety.

The development of antiviral drugs for SARS-CoV-2 mostly focuses on targeting the viral entry process or the viral genome replication.^4^ An essential step of SARS-CoV-2 genome replication requires two viral proteases, main protease (M^pro^) and papain-like protease, to cleave the precursor polyproteins into nonstructural proteins.^4^ Unlike any known human proteases, M^pro^ selectively cleaves polypeptides after a glutamine (Gln) residue, which makes M^pro^ a more promising target for drug development.

First, Qiao et al.^1^ designed 32 M^pro^ candidate inhibitors based on the crystal structures of M^pro^ and co-crystal structures of M^pro^ in complex with two anti-hepatitis C virus drugs (boceprevir and telaprevir). S1’, S1, S2, and S4 are the active sites of SARS-CoV-2 M^pro^.^5^ The peptidomimetic inhibitors were designed with three fragments (P1, P2, and P3) using the following strategy. To ensure the antiviral activity, P1 contains an aldehyde as a warhead to form a covalent bond with M^pro^ catalytic site Cys145 and a γ-lactam derivative of Gln to occupy the S1 site. To increase in vivo exposure, P2 has a bicycloproline moiety from either boceprevir or telaprevir to occupy the S2 site. To enhance the property of potency and pharmacokinetics (PK), P3 has various hydrophobic subgroups to occupy the S4 site. Finally, 32 compounds (MI-01–MI-32) with the abovementioned P1, P2 of boceprevir or telaprevir, and various P3 fragments were synthesized and characterized by nuclear magnetic resonance (NMR) and electrospray ionization mass spectrometry (ESI*-*MS).

Next, Qiao et al.^1^ evaluated the inhibitory activities and binding abilities of these compounds to M^pro^ by fluorescence resonance energy transfer (FRET) and differential scanning fluorimetry (DSF). All 32 compounds showed potent inhibition (IC_50_ = 7.6−748.5 nM) and tight binding to M^pro^. As expected, all P1, P2, and P3 fragments of MI-23, one of the most active compounds, respectively occupy the S1, S2, and S4 sites of M^pro^ well, and the warhead of MI-23 forms a covalent bond with Cys145 of M^pro^ by crystal structure analysis.

**Fig. 1:**
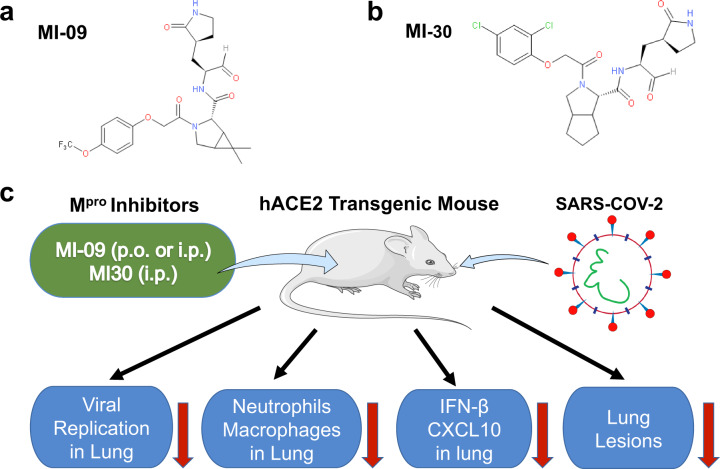
Anti-SARS-CoV-2 efficacy of M^pro^ inhibitors in a hACE2 transgenic mouse model. (**a**, **b**) Chemical structures of MI-09 and MI-30. **c** MI-09 (i.p. or p.o.) and MI-30 (i.p.) treatments inhibit SARS-CoV-2 viral replication, suppress SARS-CoV-2-induced infiltration of immune cells (neutrophils and macrophages) and secretion of IFN-β and CXCL10 in lung tissues, and reduce SARS-CoV-2-induced lung lesions in hACE2 transgenic mice

To select the best candidate inhibitors for animal experiments, Qiao et al.^1^ further screened 20 compounds (IC_50_ < 50 nM) using CCK8 assay, RT-qPCR, and cell protection assay in a spectrum of cell lines (Vero E6, HPAEpiC, LO2, BEAS-2B, A549, and Huh7). Six compounds exhibited more cell protection than other compounds and two known M^pro^ inhibitors (GC376 and 11b). Then, they studied the PK and toxicity of MI-09 and MI-30 (Fig. 1a, b), the two best candidates, in rats. The two compounds showed good PK properties and did not display noticeable toxicity in animals at the doses tested. Of notice, MI-09 and MI-30 showed oral bioavailability of 11.2% and 14.6%, respectively, suggesting the potential for the development of oral drugs.

Finally, Qiao et al.^1^ assessed the in vivo antiviral activities of MI-09 and MI-30 in a hACE2 transgenic mouse model. Mice were challenged with SARS-CoV-2 (5 × 10^6^ TCID_50_ virus/mouse mimicking moderate infection), and then treated orally (p.o.) or intraperitoneally (i.p.) with MI-09 (i.p. or p.o.), MI-30 (i.p.), or the vehicle (control), for 5 days. On 3 days post-infection (dpi), all compound-treatments decreased viral loads in lung tissues compared to the vehicle treatment. Histopathologically, all compounds-treated mice displayed slighter alveolar septal thickening and milder inflammatory cell infiltration than the controls. Furthermore, all compound-treatments reduced the expression of interferon-beta (IFN-β), and C-X-C motif chemokine ligand 10 (CXCL10), and the occurrence of neutrophils and macrophages in the lungs compared with the vehicle treatment. The results indicate that p.o. or i.p. treatment with the M^pro^ inhibitors is able to inhibit SARS-CoV-2 replication and reduce SARS-CoV-2-induced lung lesions in vivo.

In summary, Qiao et al.^1^ designed and synthesized 32 new peptidomimetic compounds targeting SARS-CoV-2 M^pro^, and characterized them thoroughly by diverse in vitro and in vivo approaches. Given the urgent need for novel agents to fight against COVID-19, this work is timely and important. This is the first study to demonstrate that p.o. or i.p. treatment with SARS-CoV-2 M^pro^ inhibitors shows promising efficacy against SARS-CoV-2 infection in a hACE2 transgenic mouse model (Fig. 1c). The new inhibitors were designed to exclusively target SARS-CoV-2 M^pro^ rather than human proteases.^1^ Whether they do have fewer side effects on COVID-19 patients remains to be determined. Also, both MI-09 and MI-30 display more than 10% oral bioavailability, but the half-life of these compounds (p.o. 20 mg/kg) is less than 1 h in rats.^1^ Further research is warranted to improve their bioavailability and PK property, to develop them as oral anti-SARS-CoV-2 drugs.
